# *Pseudouroctonus maidu*, a new species of scorpion from northern California (Scorpiones, Vaejovidae)

**DOI:** 10.3897/zookeys.584.6026

**Published:** 2016-04-25

**Authors:** Warren E. Savary, Robert W. Bryson Jr.

**Affiliations:** 1 Department of Entomology, California Academy of Sciences, 55 Music Concourse Drive, Golden Gate Park, San Francisco, CA 94118, USA; 2 Department of Biology & Burke Museum of Natural History and Culture, University of Washington, Box 351800, Seattle, WA 98195-1800, USA

**Keywords:** California, *Kovarikia*, *Pseudouroctonus*, taxonomy, Vaejovinae

## Abstract

A new species of vaejovid scorpion from northern California, *Pseudouroctonus maidu* **sp. n.**, is named and described. This new species appears to be most similar to *Pseudouroctonus iviei* (Gertsch & Soleglad, 1972) and *Pseudouroctonus glimmei* (Hjelle, 1972).

## Introduction

Recent fieldwork in northern California has revealed the presence of a previously undescribed species in the vaejovid scorpion genus *Pseudouroctonus* Stahnke, 1974. To facilitate its inclusion in discussions of ongoing systematic and phylogeographic studies of *Pseudouroctonus* and its near relatives (Francke and Savary 2006, Bryson et al. 2013, Bryson et al. 2014, and others in preparation), the new species is named and described herein. It represents the third species of *Pseudouroctonus* in California, all endemic to the state, and only the fourth new species of scorpion to be described from California in the past twenty years.

## Materials and methods

Study specimens were preserved in 70% ethanol and examined and photographed at 6× to 30× magnification with a Wild MP5 stereo microscope. Specimens were photographed under ultraviolet light following Volschenk (2005) for illustrative purposes. Terminology for carination and hemispermatophore follows Williams (1980); trichobothrial terminology follows Vachon (1974). Measurements cited are standard ones used in scorpion systematics, as defined by Williams (1980), unless otherwise noted.

## Taxonomy

### Family Vaejovidae Thorell, 1876 Subfamily Vaejovinae Thorell, 1876 Genus *Pseudouroctonus* Stahnke, 1974

#### Pseudouroctonus maidu sp. n.

Taxon classificationAnimaliaScorpionesVaejovidae

http://zoobank.org/A7132001-9B73-4DD5-ABDB-2F05CDB235EE

Figs 1 , 2–7 , 8–10 , 11–12 , 13–16 , 17–18 , 19

##### Type material.

Holotype: Adult ♀ [CASENT 9057357]. Hwy 49 between Auburn and Cool, 1.6 km SE confluence of North and Middle Forks of the American River, El Dorado Co., California (38°54'35.78"N, 121°1'40.66"W; 352 m elevation). 23 September 2013 (R. W. Bryson Jr.). Paratypes: Same locality as holotype, 23 September 2013 (R. W. Bryson Jr.), 2 ♂, 4 ♀ [CASENT 9057358]. Additional material: Same locality as holotype, 6 May 2013 (R. W. Bryson Jr.), 1 ♀ [CASENT 9057358].

##### Additional material examined.

*Pseudouroctonus iviei*: USA: California: Butte County – Pulga Rd nr junction with Hwy 70, above North Fork Feather River, 23 September 2013 (R. W. Bryson Jr.), 1 ♀ [CASENT]; El Dorado County – Hwy 49 between Auburn and Cool, 1.6 km SE confluence of North and Middle Forks of the American River, 6 May 2013 (R. W. Bryson Jr.), 1 ♂ [CASENT]; Ice House Rd, 0.2 mi N junction US 50, 5 May 2013 (R. W. Bryson Jr.), 1 ♂ [CASENT]; Nevada County – San Juan, 15 September 1963 (J. Ivie, W. Ivie), ♀ holotype [AMNH]; Napa County – 0.8 miles N of Robert L. Stevenson State Park, approximately 7 miles N of Calistoga on Highway 29 in log under bark, mixed sclerophyll-conifer community dominated by Ponderosa Pine, 20 April 1968 (E. Bergmark), 1 ♀ [CASENT 9057899]; Shasta County – 200 meters from Bald Mountain Creek, next to The Nature Conservancy McCloud River Preserve, elevation 720 m (2350’), 7 August 1991 (L. H. Simons), 2 ♂ [CASENT 9057878, CASENT 9057898]; ibid., 12 August 1991 [CASENT 9057884], 1 ♂; ibid., 6 September 1991, 1 ♂ [CASENT 9057901]; ibid., 15 September 1991, 1 ♂ [CASENT 9057893]; ibid., 7 September 1991, 1 ♂; ibid., 24 July 1992, 1 ♀ [CASENT 9057897]; Tehama County – 5 meters from Dye Creek upstream from the pond at The Nature Conservancy’s Dye Creek Preserve compound, elevation approximately 110 meters (362 feet), 30 July 1992 (L. H. Simons), 1 ♂ [CASENT 9057886]; 1 meter from Dye Creek upstream from the pond at The Nature Conservancy’s Dye Creek Preserve compound, elevation approximately 110 meters (362 feet), 28 August 1992 (L. H. Simons), 3 ♂, 1 ♀ [CASENT 9057881]; 25 meters from Dye Creek upstream from the pond at The Nature Conservancy’s Dye Creek Preserve compound, elevation approximately 110 meters (362 feet), 17 April 1992 (L. H. Simons), 1 immature [CASENT 9057900]; ibid., 28 August 1992 (L. H. Simons), 3 ♂ [CASENT 9057887]; ibid., 30 July 1992 (L. H. Simons), 1 ♀ [CASENT 9057895]; ibid., 9 May 1992 (L. H. Simons), 1 immature [CASENT 9057888]; ibid., 23 May 1992 (L. H. Simons), 1 ♀, 1 immature [CASENT 9057891]. *Pseudouroctonus glimmei*: USA: California: Colusa County – Mendocino National Forest, North Fork Campground, under rocks 39°23'N, 122°39'W, elevation 460 meters, 24 February 1997 (J. Schweikert), 1 ♂, 1 ♀ [CASENT 9057862]; between Sites and Maxwell, rocks, 1 June 1966 (K. E. Lucas), 1 immature [CASENT 9057896]; Lake County – approximately 5 miles N of Rayhouse Road at junction of Cache and Davis Creeks, elevation 900 feet, 9 August 1969 (J. T. Hjelle, T. Farris), 1 ♂, 1 ♀, 1 immature [CASENT 9057867]; ibid., 15 June 1969 (J. T. Hjelle, M. Bolander), 3 ♂, 3 ♀ [CASENT 9057877]; Marin County – Bootjack Camp, Mt. Tamalpais, 10 March 1968 (K. E. Lucas), 1 ♀ [CASENT 9057894]; Mendocino County – 13 miles E of Covelo, 30 August 1969 (J. T. Hjelle, B. E. Proeres), 1 ♂ [CASENT 9057868]; 3 miles up Robinson Creek Road, off Highway 253, elevation 1000 feet, 28 August 1969 (J. T. Hjelle, B. E. Proeres), 1 ♂ [CASENT 9057883]; Napa County – .25 miles S of Lake County line on Butts Canyon Road, elevation 750 meters, 11 September 1969 (J. T. Hjelle, T. K. Glimme), 1 ♂ [CASENT 9057890]; Stanislaus County – Frank Raines Park, 18 miles W of Patterson, 27 September 1969 (S. C. Williams, J. T. Hjelle, M. M. Bentzien, W. E. Azevedo), 1 ♀.

##### Comparative diagnosis.

**Figure 1.**
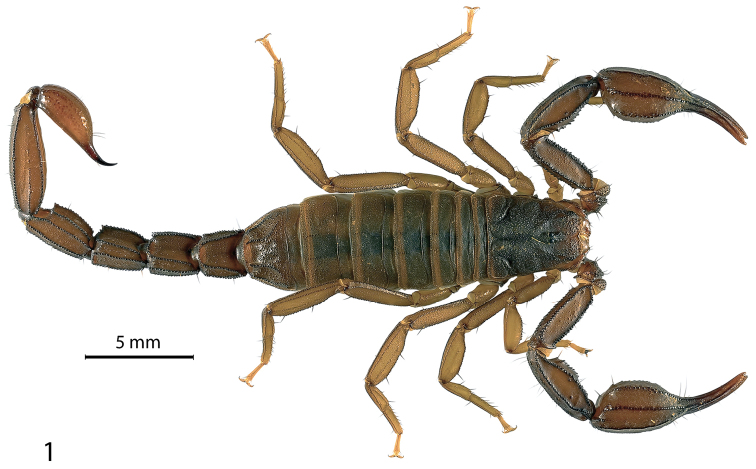
Adult female *Pseudouroctonus maidu* sp. n. in life, dorsal view.

**Figures 2–7.**
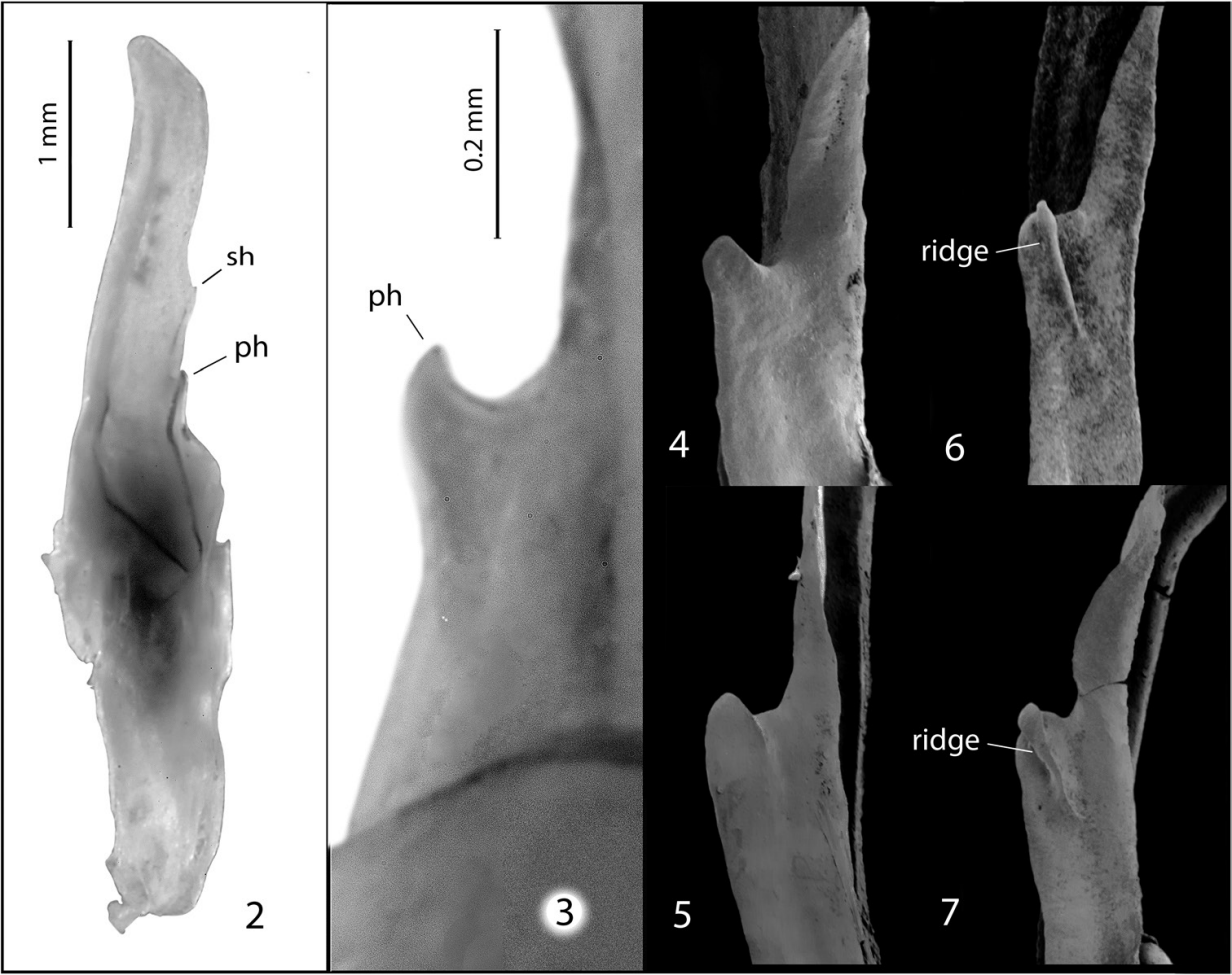
Hemispermatophores of California species of *Pseudouroctonus* Stahnke, 1974 and *Kovarikia* Soleglad, Fet & Graham, 2014: **2** hemispermatophore of *Pseudouroctonus maidu* sp. n. (ph = primary hook; sh = secondary hook) **3** primary lamellar hook of *Pseudouroctonus maidu* **4** primary lamellar hook of *Pseudouroctonus iviei* (Gertsch & Soleglad, 1972) **5** primary lamellar hook of *Pseudouroctonus glimmei* (Hjelle, 1972) **6** primary lamellar hook of *Kovarikia bogerti* (Gertsch & Soleglad, 1972) **7** primary lamellar hook of *Kovarikia angelena* (Gertsch & Soleglad, 1972).

**Figures 8–10.**
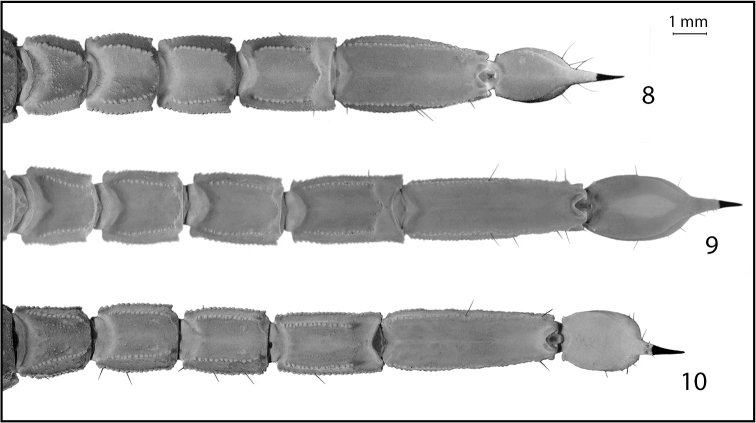
Metasomas of California species of *Pseudouroctonus* Stahnke, 1974: **8** female *Pseudouroctonus iviei* (Gertsch & Soleglad, 1972) **9** female *Pseudouroctonus glimmei* (Hjelle, 1972) **10** female *Pseudouroctonus maidu* sp. n.

**Figures 11–12.**
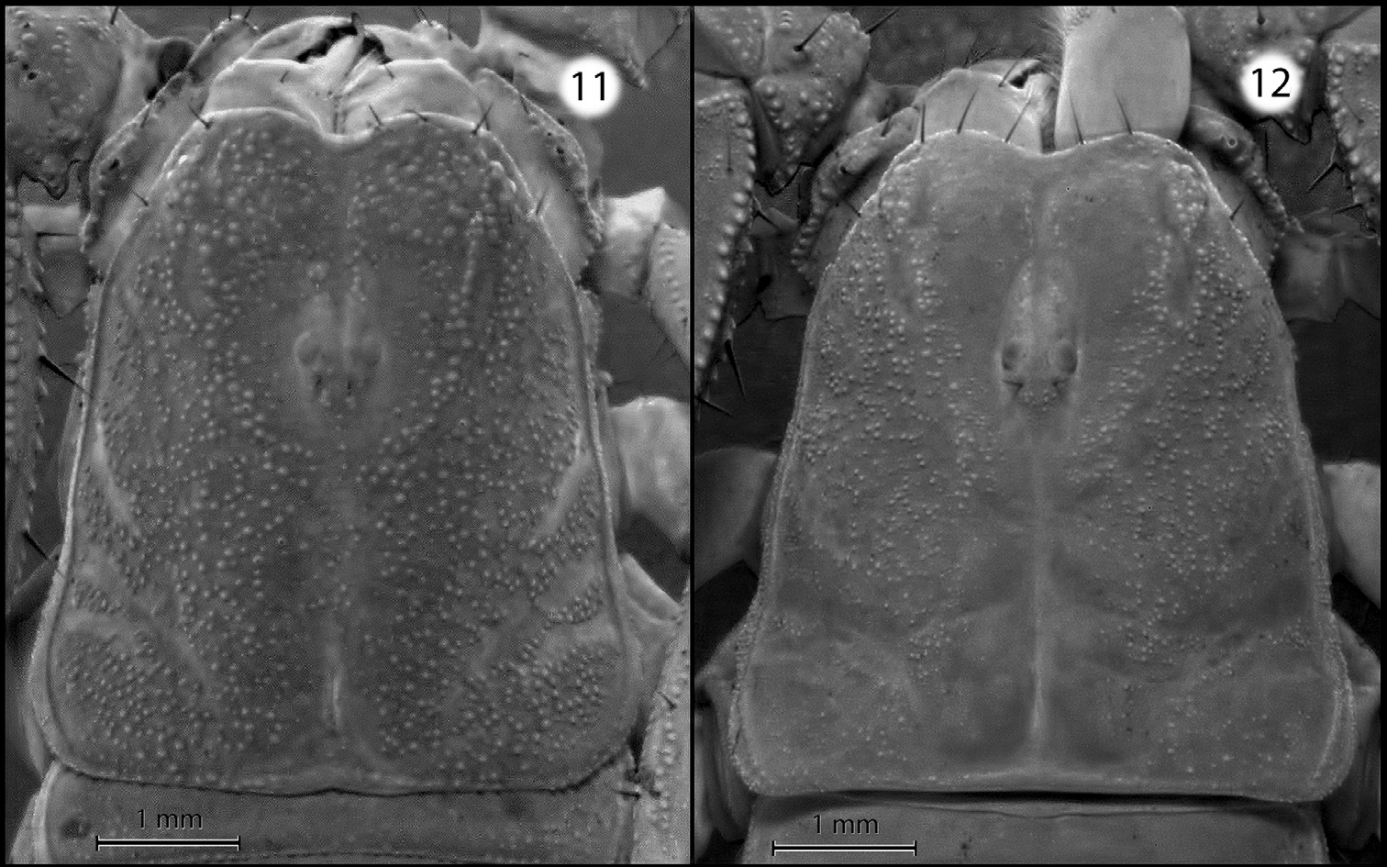
Carapaces of *Pseudouroctonus maidu* sp. n. and *Pseudouroctonus glimmei* (Hjelle, 1972): **11** female *Pseudouroctonus maidu* **12** female *Pseudouroctonus glimmei*.

**Figures 13–16.**
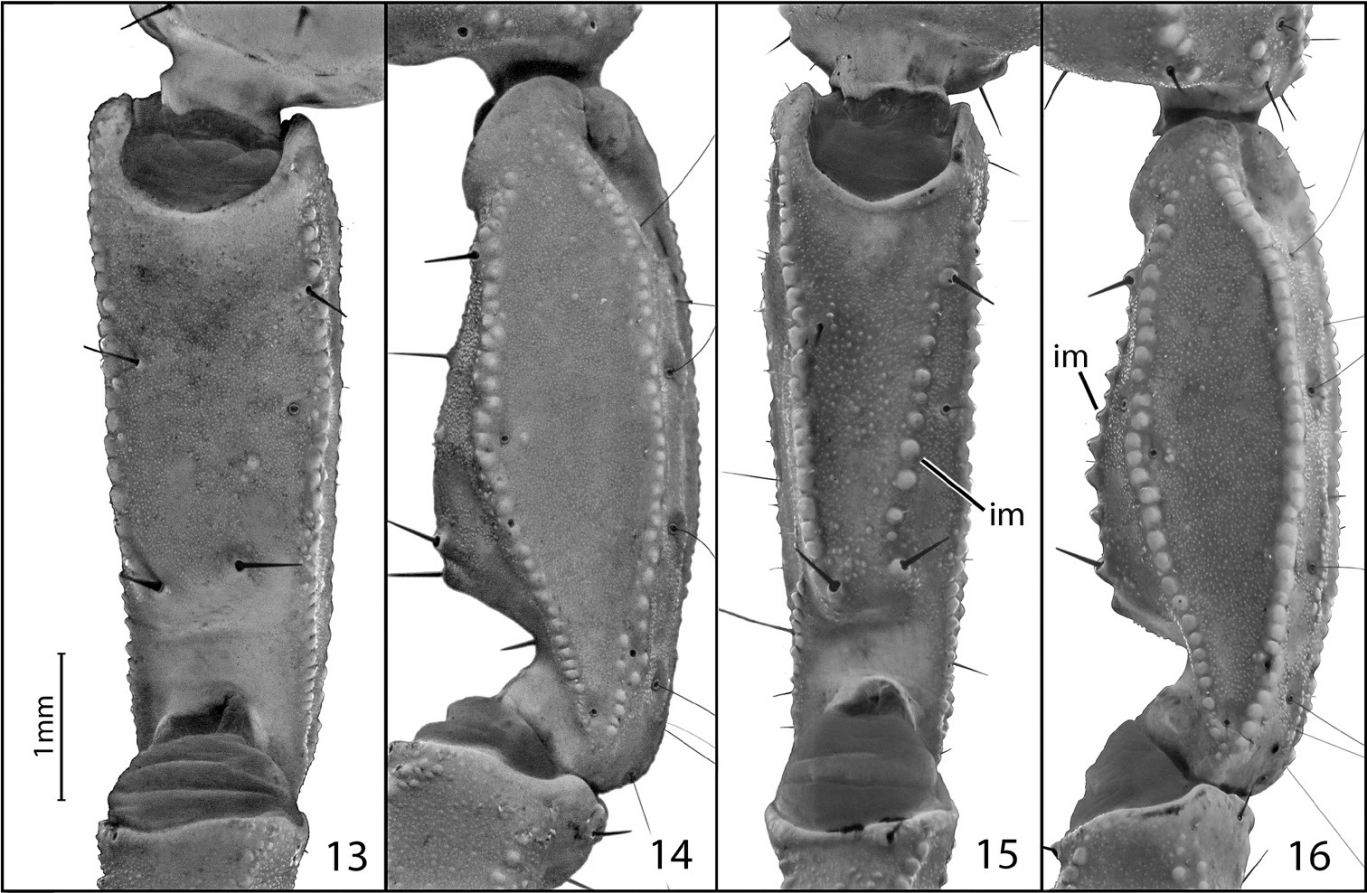
Pedipalp patellas of *Pseudouroctonus glimmei* (Hjelle, 1972) and *Pseudouroctonus maidu* sp. n.: **13** female *Pseudouroctonus glimmei*, prolateral (internal) view **14** *Pseudouroctonus glimmei*, dorsal view **15** female *Pseudouroctonus maidu*, prolateral (internal) view **16** *Pseudouroctonus maidu*, dorsal view. The internomedial carina is indicated by “im”.

Members of this small to medium-sized, darkly pigmented species (Fig. 1) appear to be most closely related to *Pseudouroctonus iviei* (Gertsch & Soleglad, 1972) and *Pseudouroctonus glimmei* (Hjelle, 1972), which together form a distinct group within the genus. All three species can be distinguished from other *Pseudouroctonus* by the lack of a distinct ridge on the primary lamellar hook of the hemispermatophore (Figs 2–7; see also figs 28 and 29 in Williams and Savary 1991). *Pseudouroctonus maidu* differs from *Pseudouroctonus iviei* in having a proportionately less elongate and significantly less inflated fifth metasomal segment and in having the second metasomal segment longer than wide (see Figs 8–10 and Table 1). *Pseudouroctonus maidu* differs from *Pseudouroctonus glimmei* in having a more granular carapace (Figs 11 and 12), and in having a well-developed, coarsely granular internomedial carina on the pedipalp patella (Figs 13–16).

**Table 1.** Morphological measurements (in millimeters) and meristic counts of *Pseudouroctonus maidu* sp. n.

	Holotype (female)	Paratype (female)	Paratype (female)	Paratype (female)	Paratype (female)	Paratype (male)	Paratype (male)
Total Length	40.53	39.01	37.68	38.59	36.06	35.19	31.40
Carapace Length	4.93	4.80	4.67	4.67	4.60	4.33	3.87
Carapace Width at lateral eyes	2.60	2.60	2.47	2.47	2.40	2.47	2.00
Carapace Width at median eyes	3.53	3.60	3.27	3.27	3.20	3.20	2.73
Carapace Width at posterior edge	4.20	4.27	4.00	4.27	3.93	3.87	3.33
Depth of median notch	0.20	0.17	0.17	0.17	0.17	0.17	0.07
Carapace anterior margin to median eyes	1.73	1.67	1.57	1.63	1.57	1.50	0.60
Diameter of median eye	0.17	0.20	0.17	0.20	0.17	0.20	0.10
Distance between median eyes	0.23	0.30	0.30	0.27	0.23	0.27	0.10
Number of lateral eyes	3/3	3/3	3/3	2/2	2/3	3/3	2/2
Mesosoma Length	12.33	11.47	11.27	11.47	11.00	9.33	9.00
Metasoma Length	23.27	22.74	21.74	22.45	20.46	21.53	18.53
Metasoma segment I Length	2.47	2.40	2.33	2.33	2.27	2.27	2.07
Metasoma segment I Width	2.53	2.40	2.33	2.33	2.27	2.27	2.00
Metasoma segment I Depth	2.13	2.00	2.00	2.00	1.93	1.80	1.73
Metasoma segment II Length	2.73	2.67	2.67	2.53	2.60	2.60	2.20
Metasoma segment II Width	2.27	2.27	2.27	2.20	2.13	2.20	1.87
Metasoma segment II Depth	2.07	2.00	1.93	1.93	1.87	1.80	1.67
Metasoma segment III Length	2.87	2.87	2.67	2.73	2.73	2.73	2.33
Metasoma segment III Width	2.20	2.13	2.13	2.20	2.07	2.07	1.87
Metasoma segment III Depth	2.07	2.00	1.93	1.87	1.87	1.93	1.67
Metasoma segment IV Length	3.67	3.60	3.27	3.60	3.33	3.33	2.80
Metasoma segment IV Width	2.07	2.07	2.13	2.07	1.93	2.00	1.73
Metasoma segment IV Depth	1.87	2.00	1.73	1.80	1.73	1.80	1.60
Metasoma segment V Length	5.73	5.53	5.33	5.73	4.13	5.40	4.73
Metasoma segment V Width	2.00	2.00	2.00	2.00	1.80	1.87	1.73
Metasoma segment V Depth	1.80	1.73	1.73	1.73	1.67	1.67	1.60
Telson Length	5.80	5.67	5.47	5.53	5.40	5.20	4.40
Vesicle Length	3.53	3.40	3.47	3.47	3.33	3.20	3.00
Vesicle Width	2.07	1.93	2.00	1.93	1.87	1.80	1.60
Vesicle Depth	1.67	1.53	1.60	1.53	1.60	1.53	1.40
Aculeus Length	1.93	1.80	1.80	1.67	1.67	1.73	1.33
Pedipalp Length	17.67	16.94	16.40	16.6	16.60	15.96	13.41
Pedipalp Femur Length	4.40	4.27	4.13	4.27	4.07	4.00	3.27
Pedipalp Femur Width	1.53	1.47	1.40	1.40	1.47	1.40	1.13
Pedipalp Femur Depth	1.27	1.27	1.20	1.20	1.27	1.00	1.00
Pedipalp Patella Length	4.87	4.60	4.47	4.53	4.53	4.43	3.67
Pedipalp Patella Width	1.80	1.67	1.60	1.80	1.73	1.53	1.33
Pedipalp Patella Depth	1.47	1.47	1.40	1.40	1.53	1.27	1.13
Pedipalp Chela Length	8.40	8.07	7.80	7.80	8.00	7.53	6.47
Palm Length	4.67	4.67	4.53	4.60	4.33	4.33	3.60
Palm Width	2.60	2.40	2.47	2.33	2.27	2.27	1.93
Pedipalp Chela Depth	2.53	2.60	2.47	2.40	2.60	2.60	2.33
Fixed Finger Length	3.60	3.40	3.33	3.33	3.53	3.33	2.60
Movable Finger Length	4.67	4.47	4.47	4.27	4.33	4.20	3.33
Supernumeraries FF	6	6	6	6	6	6	6
Rows FF	6	6	6	6	6	6	6
Supernumeraries MF	7	7	7	7	7	7	7
Rows MF	6	6	6	6	6	6	6
Number of Pectinal Teeth	10/10	11/10	11/11	11/11	11/11	11/11	11/11

Unlike other members of *Pseudouroctonus*, *Pseudouroctonus maidu*, *Pseudouroctonus iviei* and *Pseudouroctonus glimmei* bear an elevated secondary lamellar hook on the hemispermatophore (Figs 2–5; see also figs 26 through 29 in Williams and Savary 1991). This feature is also present in members of the related genus *Kovarikia* Soleglad, Fet & Graham, 2014 from southern California, although members of that genus also exhibit a distinct ridge on the primary lamellar hook of the hemispermatophore (Figs 6 and 7; see also figs 22 through 27 in Williams and Savary 1991) that is lacking in *Pseudouroctonus maidu*, *Pseudouroctonus iviei* and *Pseudouroctonus glimmei*.

##### Description.

Based on the adult holotype female [CASENT 9057357]. *Color*: Base color uniform dark reddish brown with legs, chelicerae and underside of preabdomen slightly paler.

**Figures 17–18.**
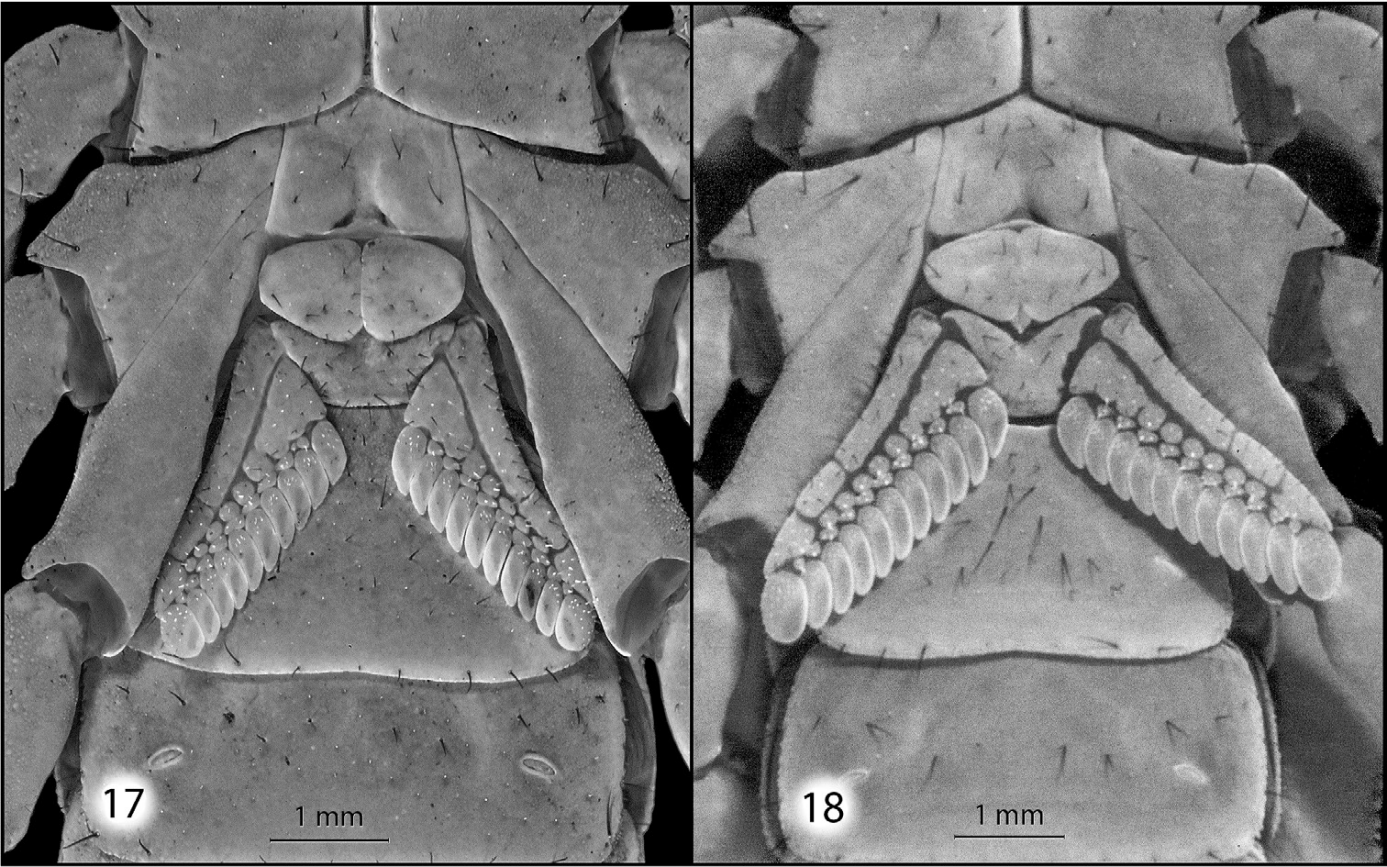
Sternum and pectines of *Pseudouroctonus maidu* sp. n.: **17** female **18** male.

**Figure 19.**
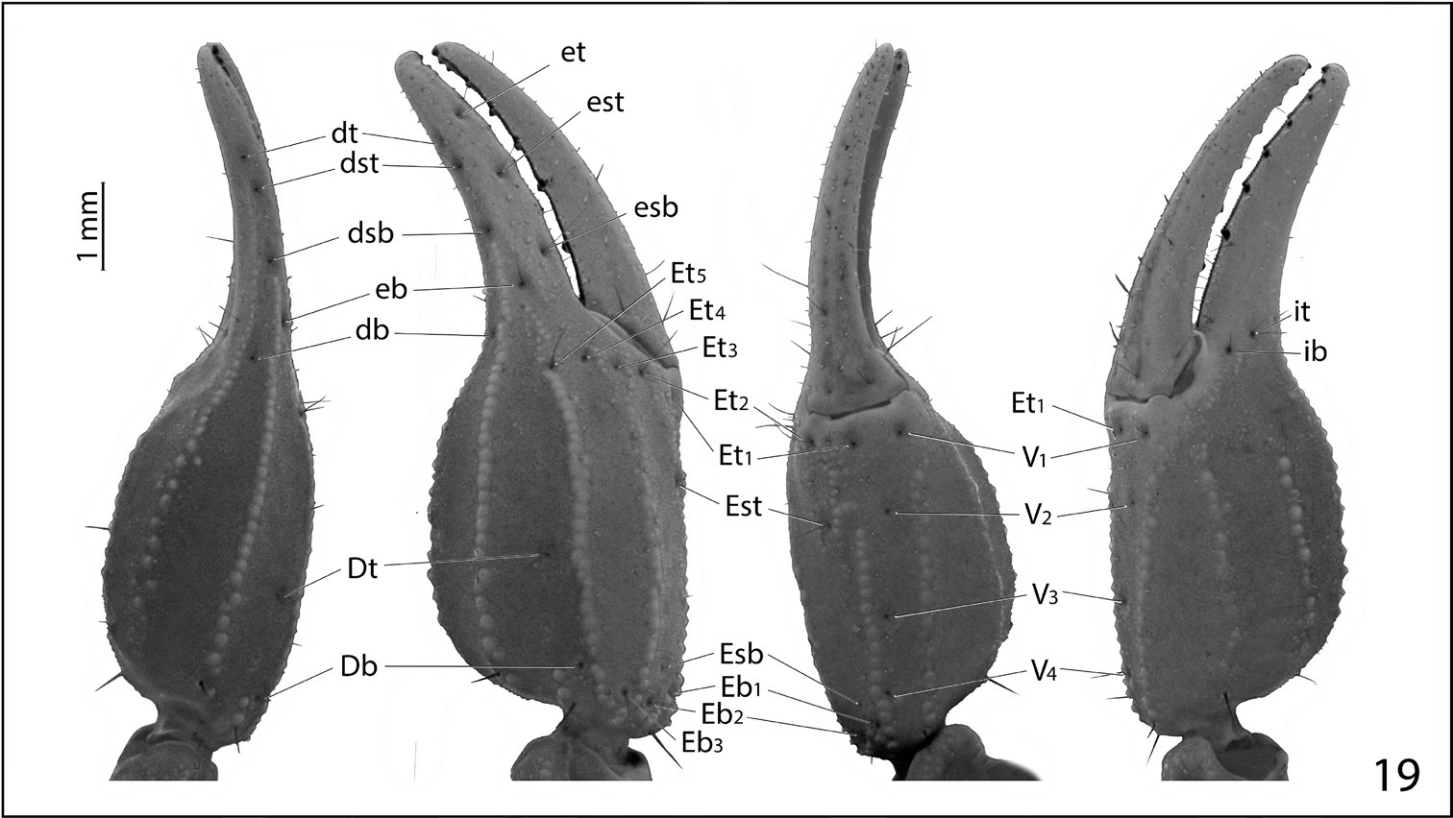
Trichobothrial map of pedipalp chela of female *Pseudouroctonus maidu* sp. n.

*Morphology*: Carapace (Fig. 11): Longer than wide. Median eyes on anterior 35%. Ocular tubercle low, without superciliary crests. Median eyes 0.2 mm in diameter. Three pairs of lateral eyes, posterior-most smallest. Anterior median furrow narrow and distinct. Posterior median furrow shallow, less distinct. Anterior margin broadly bilobed, with 3 pairs of setae. Surface densely granular throughout, with granulation extending into the median furrow. Tergites: Coarsely granular, particularly on lateral and posterior margins. Tergite VII with four well-developed carinae. Sternum (Fig. 17): Subpentagonal, slightly broader than long; median longitudinal furrow deep; with 8 setae (nearly symmetrical in arrangement). Genital opercula (Fig. 17): Well-developed, with ten visible setae or setal sockets each. Pectines (Fig. 17): With 6/7 middle lamellae, of which the proximal appear to be partially fused; 10 teeth on each side, the most distal of which is weakly expanded laterally. Sternites: Anterior (stigmata-bearing) sternites I–VI very finely granular. Sternite VII more coarsely granular, with one pair of weakly developed longitudinal carinae. Stigmata small, elongate, and suboval in shape, about twice as wide as long. Metasoma (Fig. 10): Dorsolateral carinae on I–V strong, denticulate/serrate. Lateral supramedian carinae on I–IV strong, denticulate/serrate. Lateral inframedian carinae on I strong, granular, nearly complete; on II-IV absent. Lateral median carina on V granular on proximal half, obsolete distally. Ventrolateral carinae on I–V, ventral submedian carinae on I–IV and ventral median carina on V strong, denticulate/serrate. Setation on I–IV: Dorsolaterals 0,0,0,1; lateral supramedian 0,1,1,1; lateral inframedian 1,0,0,0; ventrolateral 2,2,2,3; ventral submedian 2,3,3,3. Setation on V: Dorsolateral 2, lateromedian 2, ventrolateral 3 and ventromedian 4. Intercarinal spaces finely granular. Telson (Fig. 10): Vesicle robust, slightly wider than fifth metasomal segment; rugose ventrally, sparsely setose. Aculeus without basal patches of microdenticles. Chelicera: Fixed finger shorter than chela width, movable finger shorter than chela length. Chela with 3 setae dorsally. Fixed finger with basal bicusp nearly symmetrical (distal cusp slightly larger); ventral margin lacking accessory denticles. Movable finger with two dorsal subdistal teeth; with a distinct serrula ventrally, and with ventral carina smooth and strongly delineated. Pedipalp femur: Dorsointernal, externomedial, dorsoexternal, ventrointernal, and internomedial carinae strong, coarsely granular; ventroexternal carina obsolete. Orthobothriotaxia “C”. Internal face with three submedian setae; external face with 3 setae along externomedian keel; all surfaces with moderately dense granulation. Pedipalp patella (Figs 15–16): Dorsointernal, dorsoexternal, ventrointernal, and ventroexternal carinae strong, coarsely granular; internomedian carina well-developed and granular, extending nearly entire length of patella. Dorsal externomedian carina strong, complete, and granular; ventral externomedian carina weaker, with several interruptions, and not extending entire length of patella. Intercarinal spaces finely granular. Orthobothriotaxia “C”. Pedipalp chela: Carinae granulose; intercarinal spaces very finely granular. Fixed finger with 6 rows of granules and 6 inner accessory denticles; movable finger with 6 rows of granules and 7 inner accessory denticles. Orthobothriotaxia “C” (Fig. 19), with trichobothrium *Dt*, located near the midpoint of the chela, conspicuously closer to trichobothrium *Est* than to the palm base, trichobothria *Et_1_* and *V_1_* approximately equidistant from the articulation of the movable chela finger, and 4 trichobothria in the ventral series. Walking legs: Typical of the genus. Prolateral and retrolateral pedal spurs present on all legs; tibial spurs absent. Ventral surface of tarsus with single median row of spinules, this terminating distally in two pairs of spinules (one prolateral pair and one retrolateral pair). Unguicular spine well-developed and pointed.

*Measurements*: See Table 1.

**Male.** Similar to female. Genital papillae well-developed, conspicuous (Fig. 18). Hemispermatophore with secondary lamellar hook (Fig. 2); primary hook without raised ridge (Figs 2–7); mating plug present; similar to other members of genus in all other respects.

**Variation.** See Table 1. The pectinal tooth count was 11/11 in both examined males, and ranged from 10/10 to 11/11 in examined females (n=5).

##### Etymology.

Named after the Maidu people of northern California, in whose historic lands the species occurs.

##### Distribution.

**Figure 20.**
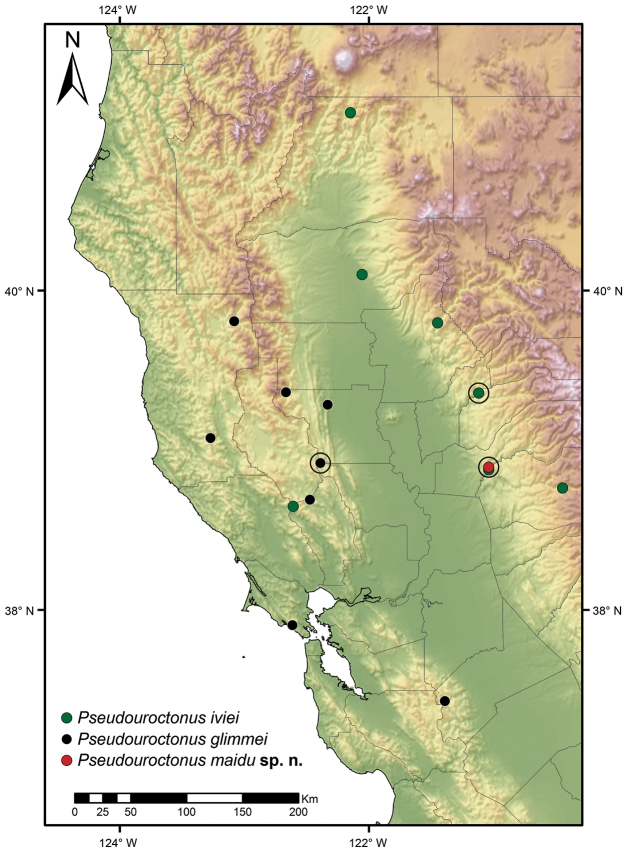
Localities of *Pseudouroctonus iviei* (Gertsch & Soleglad, 1972), *Pseudouroctonus glimmei* (Hjelle, 1972), and *Pseudouroctonus maidu* sp. n., examined for this study. Circled dots represent type localities.

Known only from the type locality near the confluence of North and Middle Forks of the American River in El Dorado County, California (see Fig. 20).

##### Natural history.

Specimens were collected on 6 May 2013 and 23 September 2013. Two were found during the day on 6 May beneath rocks in moist leaf litter along a steep rocky drainage. The remaining specimens were collected by UV detection at night on 23 September. All were found on or near the bottom of a rocky embankment next to the highway. The area is characterized by oak-dominated woodlands on the drier south-facing slopes and mixed-conifer forest on the cooler north-facing slopes. Most *Pseudouroctonus maidu* were found on north-facing slopes with scattered patches of moss-covered rocks. *Pseudouroctonus iviei* were common in drier rocky habitat with an abundance of oak leaf litter, and the two species were found in close proximity (within 0.5 m of each other) under rocks in a steep rocky drainage connecting the two types of habitat.

## Supplementary Material

XML Treatment for Pseudouroctonus maidu
